# Supplementary figures and images for: Effect of Dedifferentiation on Time to Mutation Acquisition in Stem Cell-Driven Cancers
Source: PLoS Comput Biol. 2014 Mar 6;10(3):e1003481. doi: 10.1371/journal.pcbi.1003481 (PMC3945168; doi:10.1371/journal.pcbi.1003481)

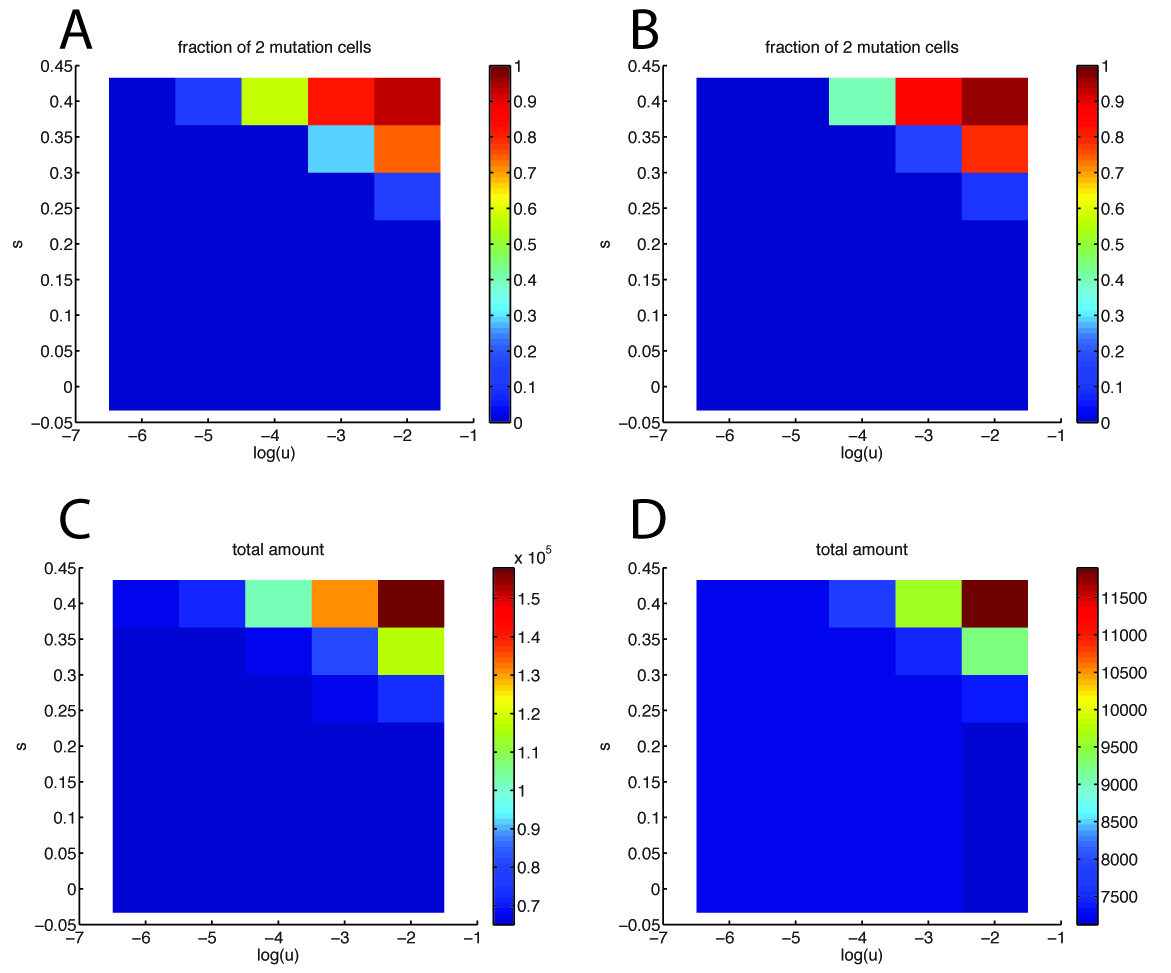

Supplement: Figure S3 — Steady-state progenitor distributions in the absence of stem cell mutation but with progenitor competition. Top: The fraction of mutant cells as a function of mutation rate and proliferative advantage for (A,C) local (age-dependent) competition between subpopulations given by Eq. (S7), and (B,D) global competition between subpopulations given by Eq. (S8). Bottom: Corresponding plots of total cell density. Basal dynamics are constant death rate and sigmoidal birth rate with maximal growth rate , for . The same carrying capacity is used for all simulations: , , . Note that there is a sharp transition zone at which mutant cells go from nearly zero fraction of total population to majority of the differentiating cell population. However, the mutation rate and proliferative advantage at which this is observed is unreasonably high, just as for the model without progenitor competition (Fig. 2). (TIF) [file pcbi.1003481.s003.tif]
