# Supplementary material for: Effect of Dedifferentiation on Time to Mutation Acquisition in Stem Cell-Driven Cancers
Source: PLoS Comput Biol. 2014 Mar 6;10(3):e1003481. doi: 10.1371/journal.pcbi.1003481 (PMC3945168; doi:10.1371/journal.pcbi.1003481)
Supplement: Text S1 — Analytic solutions and derivations, alternative models, and Matlab code. (PDF) [file pcbi.1003481.s006.pdf]

Supporting Text for ‘A Hybrid Deterministic-Stochastic Model for Mutation Acquisition in Stem Cell Driven Cancers’

Alexandra Jilkine[1,2], Ryan Gutenkunst[1*]
1 Molecular and Cellular Biology, University of Arizona, Tucson, AZ, USA
∗ rgutenk@email.arizona.edu

Progenitor cells

Analytic solution for progenitor model

If we neglect mutation, each subpopulation of progenitor cells is governed by a single PDE (Eq. (1)), which can be solved by the method of characteristics. Without loss of generality, assume

$$\frac{da}{dt} = 1 \text{ on } \frac{dp_i}{dt} = (\sigma(a) - \mu(a))p_i.$$

For $a > t_0$ the solution is

$$p_i(a, t_0) = p(a_0, 0)e^{\int_{a_0}^{a}(\sigma(s)-\mu(s))ds}. \tag{S1}$$

The behavior of this solution is determined by the initial condition $p_i(a_0, 0)$. For $a < t_0$ the solution is

$$p_i(a, t_0) = p_i(0, t_0)e^{\int_0^{a_0}(\sigma(s)-\mu(s))ds}, \tag{S2}$$

which results in

$$p_i(a, t) = p_i(0, t - a)e^{\int_0^{a}(\sigma(s)-\mu(s))ds}, \quad a < t. \tag{S3}$$

Because we are interested in long term behavior, we are only concerned with the solution for $t > a$, which is determined by the boundary condition.

For the stem cell boundary condition (Eq. (3)), the boundary condition does not depend on the state of the population at a previous time, and for $t > a$ equation (S3) becomes

$$p_i(a, t) = (2\alpha_{D,i} + \alpha_{A,i})\, n_i(t - a)e^{\int_0^{a}(\sigma(s)-\mu(s))ds}. \tag{S4}$$

Note that the solution has the form $p_i(a, t) = \alpha n_i(t-a)e^{r(a)}$, where $e^{r(a)}$ is a steady state age distribution, which multiplies the boundary condition provided by the stem cells. Hence, the shape of the age-distribution is largely determined by the functional forms of the the birth and death of the differentiated cells, which are given by Eq. (5). (Alternatively Hill functions to model age-dependent proliferation/death rates does not qualitatively change the obtained results and uses the same number of parameters with identical meanings.)

Using equation (S4) it is possible to analytically calculate the the steady state age distribution

$$e^{\int_0^{a}(\sigma(s)-\mu(s))ds} = e^{r(a)}.$$

For the switch-like birth and death rates given by Eq. (5) we obtain

$$r(a) = \frac{(b-d)a}{2} - \frac{b}{2\rho_b}\left[\ln\left(\frac{\cosh(\rho_b(a-\omega_b))}{\cosh(\rho_b\omega_b)}\right)\right] - \frac{d}{2\rho_d}\left[\ln\left(\frac{\cosh(\rho_d(a-\omega_d))}{\cosh(\rho_d\omega_d)}\right)\right]. \tag{S5}$$

The progenitor equation (Eq. (1)) can be easily modified to have a maximal carrying capacity K_i for each sub-population.

$$\frac{\partial p_i}{\partial t} + \frac{da}{dt}\frac{\partial p_i}{\partial a} = \sigma(a)p_i(1 - p_i/K_i) - \mu(a)p_i. \tag{S6}$$

This does not change the qualitative nature of the solutions (See Fig. S1B,C).

Robustness to parameter variations in the progenitor model

To test which parameters in the model have the largest effect on the steady state age distribution of differentiated cells, we varied all the parameters in Eq. (5). For each parameter the age dependent growth rate $r(a) = \int_0^a (\sigma(s) - \mu(s))ds$ and the steady state age distribution $N_{sc} \times e^{r(a)}$ are plotted in Fig. S2. Note that the proliferation rate b has a great effect on the age distribution of the population. On the other hand, increasing the removal/clearance rate d does not change the maximal value of the age distribution, only the location at which the peak begins to fall off, because it speeds up the removal of older cells that are not proliferating. Increasing b two-fold, from 1 to 2, increases the maximal value of the age distribution 10,000 fold (Fig. S2B), but changing d only marginally moves the point at which the age distribution begins to fall off, and does not affect the peak value (Fig. S2D). Note that this result was obtained with the assumption that $\omega_d > \omega_b$, i.e., most cells begin to die off after they are done proliferating. We also tested the effect of shifting ω_d and ω_b. (Note that ω_b or ω_d=0 indicates that the rate is constant for all maturity levels.) A mutation that enables progenitor cells to undergo more divisions before entering senescence and apoptosis has much greater effects on population dynamics than one that enables cell removal to begin earlier. We can also conclude that enhanced cell clearance rate, whether by the immune system or other methods will not make much difference in this model if the proliferation rate is increased. Increasing the steepness of either switch (ρ_b and ρ_d) did not greatly affect the maximal value of the age distribution, but made the distribution more box-shaped (Fig. S2F,H).

Alternative Models of Progenitor Dedifferentiation and Competition

Alternative model for progenitor cells including competition

The solutions to our progenitor model in the main text are entirely determined by the dynamics of the stem cells, with no interactions between the different mutants. Here we considered an alternate model including competition between multiple progenitor cell subpopulations. Taking into account competition, our PDE system becomes

$$\frac{\partial p_i}{\partial t} + \frac{\partial p_i}{\partial a} = (1-u)\sigma_i(a)\left(1 - \frac{\sum_{j=0}^{M} p_i}{K_i}\right) n_i - \mu_i(a)p_i, \tag{S7}$$

where the local carrying capacity of the progenitors in the absence of other subpopulations is $K_i\left(1 - \frac{\mu_i}{(1-u)\sigma_i}\right)$.

We can compare system (S7) to the classic Lotka-Volterra model. Note that, in general, for a Lotka-Volterra system with multiple species and non-redundant values of reaction constants, it can be shown that there is only one stable homogeneous equilibrium with one species dominant and the other species extinct (i.e., no co-existence steady state). However, for PDE system (S7) we observe coexistence of all three populations for different growth rates. This advection mediated coexistence for competing populations has been previously described for spatial models of competing species [1], although in our model advection is a maturation process with constant velocity, and no diffusive dispersal takes place.

Note that in system (S7), competition is between individuals of the same maturity stage only. This is appropriate if cells of different maturities can be considered as different cell types or occupy different locations in the body. If the competition is between cells of all maturity stages, rather than just cells of a given age cohort, then the competition term becomes global rather than local:

$$\frac{\partial p_i}{\partial t} + \frac{\partial p_i}{\partial a} = (1-u)\sigma_i(a)\left(1 - \frac{\sum_{j=1}^{M} \int_a p_i(a,t)\,dt}{N_k}\right) n_i - \mu_i(a)p_i(a), \tag{S8}$$

Adding either local or global competition does not influence the proportion of M-mutation cells in the progenitor model (Fig. S3). We also looked at the effect of competition between differentiated cells

can have on time to cancer acquisition in the full deterministic-stochastic model. Considering either local or global competition between progenitor subpopulations does not greatly affect time to mutation acquisition in the full model (Fig. S4B). Hence, without dedifferentiation subpopulation competition in the progenitor model is not significant in altering the time to fixation of mutant in the total cell population for neutral stem cell dynamics.

Alternative model of dedifferentiation for constant N_{sc}

In the main text, we assumed that only two-mutation progenitor cells could dedifferentiate. We also considered the waiting time to fixation when all progenitor cells have a non-zero probability ε of dedifferentiating and becoming a cancer stem cell. Every T_{gen}/N_{sc} time units, a single randomly chosen stem cell j is removed and one cell i is born with probability given by

$$P(\mathbf{n} \to \mathbf{n} + \mathbf{e_i} - \mathbf{e_j}) = (1-\varepsilon)\frac{n_j}{N_{sc}}\left[\sum_{h=0}^{M} m_{h,i}\frac{n_h}{N_{sc}}\right] + \varepsilon\frac{\int_a p_i(a)\,da}{\sum_i \int_a p_i(a)\,da}, \tag{S9}$$

where $p_i(a)$ is the density of differentiated cells of age a carrying i mutations, and ε is the proportion of cells in the stem cell pool that come from dedifferentiated cells at each replication event. We also considered a model in which all progenitor cells can dedifferentiate, but dedifferentiation is weighted by proliferation rate $\sigma(a)$ of the progenitors, with faster replicating mutants being more likely to end up dedifferentiating.

$$P(\mathbf{n} \to \mathbf{n} + \mathbf{e_i} - \mathbf{e_j}) = (1-\varepsilon)\frac{n_j}{N_{sc}}\left[\sum_{h=0}^{M} m_{h,i}\frac{n_h}{N_{sc}}\right] + \varepsilon\frac{\int_a \sigma_i(a) p_i(a)\,da}{\sum_i \int_a \sigma_i(a) p_i(a)\,da}. \tag{S10}$$

These assumptions do not significantly change the distribution of waiting times for intermediate dedifferentiation rate ε (Fig. S4B). However, the waiting time for 2 mutations in this model is faster than model Ib for high values of ε, particularly for small u values (Fig. S4D).

Derivation of exponential growth rate and critical dedifferentiation rate

In our variable stem cell population size model, the expected number of two-mutation stem cells produced per stem cell reproduction event is

$$\lambda = \frac{\delta T_{gen}}{S(t)} P(t). \tag{S11}$$

A stem cell reproduction event takes place every $T_{gen}/S(t)$ time units, and the stem cell population in changes by $\Delta S = \lambda - (1-\eta)$, where η is the probability of an asymmetric division. Taking the continuum limit, we have

$$\frac{dS}{dt} = \frac{\lambda - (1-\eta)}{T_{gen}} S. \tag{S12}$$

Note that if the mean number of dedifferentiated cells λ exceeds $1-\eta$, then $S(t)$ grows exponentially at rate

$$k = \frac{\lambda - (1-\eta)}{T_{gen}}.$$

To calculate the growth rate k, recall that the two-mutation progenitor population is given by

$$p_2(a,t) = \alpha S(t-a) e^{\int_0^a r(x)dx} = \alpha S(t) e^{-ka} e^{\int_0^a r(x)dx} \tag{S13}$$

where $\alpha = 2\alpha_{D,2} + \alpha_{A,2}$. Here we are making the approximation that most stem cells carry two-mutations, which is valid once exponential growth has proceeded for some time.

It follows that

$$P(t) = \int_0^\infty p_2(a,t)\,da = \alpha S(t) \int_0^\infty e^{-ka} e^{r(a)}\,da. \tag{S14}$$

Substituting into Eq. S12, we then have

$$\frac{dS}{dt} = S(t)\left[\alpha\delta \int_0^\infty e^{-ka} e^{r(a)}\,da - \frac{(1-\eta)}{T_{gen}}\right]. \tag{S15}$$

It follows that k is given by the solution to the integral equation

$$k = \alpha\delta \int_0^\infty e^{-ka} e^{r(a)}\,da - \frac{1-\eta}{T_{gen}}, \tag{S16}$$

which always has a unique solution for k.

If

$$\int_0^\infty e^{r(a)}\,da > \frac{1-\eta}{\alpha\delta T_{gen}} \tag{S17}$$

then $k = \frac{\lambda-(1-\eta)}{T_{gen}} > 0$ and we have exponential growth of the stem cell population. This results in a minimum dedifferentiation rate (per stem cell replication event) necessary for exponential growth given by

$$\delta_{crit} = \frac{1-\eta}{\alpha T_{gen} \int_0^\infty e^{r(a)}\,da}. \tag{S18}$$

This is equivalent to the mean number of cells coming from the dedifferentiated progenitor population being given by

$$\lambda_{crit} = \frac{(1-\eta)[1 + \alpha \int_0^\infty e^{r(a)}\,da]}{\alpha \int_0^\infty e^{r(a)}\,da}. \tag{S19}$$

This threshold behavior is very similar to what one observes for the well-studied Foerster-McKendrick Equation with an integral boundary condition:

$$\frac{\partial p_i}{\partial t} + \frac{\partial p_i}{\partial a} = -\mu(a) p_i, \tag{S20}$$

$$p(t,0) = \int_0^\infty b(a)\,p(t,a)\,da. \tag{S21}$$

There, the behavior of the solution depends on a critical quantity

$$s = \int_0^\infty b(a) \exp\left(-\int_0^a \mu(s)\,ds\right)\,da.$$

The solution undergoes exponential growth if $s > 1$, and exponential decay if $s < 1$ [2]. Our model, however, has a mixed boundary condition for $p_2(t,a)$. Hence, when $\delta < \delta_{crit}$ and $k < 0$, the stochastic term in the boundary condition dominates, and we get similar behaviour as the variable $S(t)$ case with no dedifferentiation. That is, there is neither exponential growth nor decay, and the waiting time distribution to fixation is equivalent to the fixed N case with $\delta = 0$.

References

1. Lutscher F, McCauley E, Lewis M (2007) Spatial patterns and coexistence mechanisms in rivers. Theor Pop Biol 71: 267-277.

2. Murray J (2003) Mathematical Biology. Springer.

Matlab code

Model I (Constant Stem Cell Population Size)

This code simulates trajectories for model with a constant stem cell population size with and without dedifferentiation.

```
function[Tmut]=ConstantN_dedifferentiation()

%physical parameters
T=5000; %time of integration
L=20; %length of domain (age distribution of differentiated cells)


%numerical paprameters
numx = 101;   %number of grid points in x
dx = L/(numx - 1);
x = 0:dx:L;   %vector of x values, to be used for plotting
N=length(x);
dt = 0.01;
CFL=1*dt/dx;
numt =T/dt;


% matrices for output
A = zeros(numx,201);
B = zeros(numx,201);
C = zeros(numx,201);
nplot= floor(numt/100);
counter=1;

% matrices for computation
Atemp = zeros(numx,2); %normal cells
Btemp = zeros(numx,2); %mutant 1 cells
Ctemp = zeros(numx,2); %mutant 2 cells


  %biological stem cell parameters
  alpha_D=.1; %probability of symmetric differentiation
  alpha_A=0.8; %probability of asymmetric division
  Nsc=100; %total number of  stem cells
   K=3; % number of mutations cells are allowed to accumulate
  fitness=ones(1,K); %fitness of different stem cell mutants
 %fitness=[1, 1.1, 1.2];  %fitness of different stem cell mutants
  n=[Nsc, 0, 0]'; %initial number of normal and mutant stem cells
  epsilon=0.0 %fraction of differentiated cells that de-differentiate
  dediff_pop=0; %initialize de-differentiated progenitor cell population

  %specify mutation rate and initiliaze stem cell population
    u=0.01  %mutation rate
 % specify mutation matrix
```

```
 mut=eye(K);
 mut(1,1)=1-u;
  mut(1,2)=u;
  mut(2,2)=1-u;
  mut(2,3)=u;


SC=zeros(K,numt); %intialize stem cell numbers
   for jj=1:K %prob matrix of producing diff number of offspring
       Prob(jj,:)=[0.1 0.8 0.1]

   end

R1=rand(1,numt);
R2=rand(1,numt);


  % specify proliferation and death rate of differentiated cells
  beta=zeros(K,length(x));
  r=zeros(K,length(x));
  dd=1.;
b_diff=[1.5, 1.7, 1.9]; %increasing proliferation of mutant cells
%b_diff=[1.5, (1+s)*1.5, (1+s)^2*1.5] %baseline times selection
%coefficient
for i=1:K
bb(i,:)=fitness(i)*birth(x,b_diff(i),2,10);
end
mu=dd*ones(K,numx); %constant death rate for simplicity

    beta(1:end-1,:)=(1-u)*bb(1:end-1,:);
    beta(end,:)=bb(end,:);
    r=beta-mu;


     SC(:,1) =n; %set stem cell population to initial condition


%specify initial conditions
    Atemp(1:end,1) = 0;
    A(:,1) = 0;
    Btemp(1:end,1) = 0;
    B(:,1) = 0;
    Ctemp(1:end,1) = 0;
    C(:,1) = 0;

%BC at x=0 at t=0 (needs to be updated at each time step)
    Atemp(1,1)=n(1);
    Btemp(1,1)=n(2);
    ctemp(1,1)=n(3);
```

```
    total=0; %total mutated cells

nt2=0;
nt3=0;
%iterate difference equations
for j=1:numt
%     if SC(2,j)==1 && SC(2,j-1)==0
%         T2=j*dt
%         nt2=nt2+1;
%     end

     if SC(3,j)==1 && SC(3,j-1)==0
        T3=j*dt
        nt3=nt3+1;
    end
            if SC(end,j)==Nsc
               Tmut= j*dt
               for kk=1:K
               SC(kk,j+1:end)=SC(kk,j);
               end
                break
            else
        SC(:,j+1)=SC(:,j);
%stem_tot=sum(SC(:,j))

   Atemp(1,2)=(2*alpha_D+alpha_A)*SC(1,j); %BC at x=0 updated at each timestep
   Btemp(1,2)=(2*alpha_D+alpha_A)*SC(2,j); %BC at x=0 updated at each timestep
   Ctemp(1,2)=(2*alpha_D+alpha_A)*SC(3,j); %BC at x=0 updated at each timestep

    for i=2:numx %equations for aging of differentiated cells
            Atemp(i,2) = Atemp(i,1) + CFL*(Atemp(i-1,1) -Atemp(i,1))+dt.*(beta(1,i)*Atemp(i,1)
            -mu(1,i)*Atemp(i,1));
            Btemp(i,2) = Btemp(i,1) + CFL*(Btemp(i-1,1) -Btemp(i,1))+dt.*(beta(2,i)*Btemp(i,1)
            -mu(2,i).*Btemp(i,1)+u*bb(2,i)*Atemp(i,1));
            Ctemp(i,2) = Ctemp(i,1) +CFL*(Ctemp(i-1,1) -Ctemp(i,1))+dt.*(beta(3,i)*Ctemp(i,1)
            -mu(3,i).*Ctemp(i,1)+u*bb(3,i)*Btemp(i,1));
 end

          dediff_pop= floor([trapz(x,beta(1,:)'.*Atemp(:,2)) trapz(x,beta(2,:)'.*Btemp(:,2))
          trapz(x,beta(3,:)'.*Ctemp(:,2))])*eye(K);

          if dediff_pop==0
            new_pop=zeros(1,K);
        else
            new_pop= cumsum(dediff_pop)/sum(dediff_pop);
        end
    P_plus=cumsum(prob_distribution_reproduction(Nsc, K, fitness, SC(:,j), mut) );
    P_new=(1-epsilon)*P_plus+epsilon*new_pop;
    Pp=zeros(1,length(P_new)+1);
```

```
    Pp(2:end)= P_new;
    for i=1:K
        if Pp(i)<R2(j) && R2(j)<Pp(i+1)
SC(i,j+1)=SC(i,j)+1;
        end %end reproduction loop
    end
     %remove one member of population proportional to frequency of the
     %allele at prev time step
    P_minus=cumsum(SC(:,j)/Nsc);
    Pm=zeros(1,length(P_minus)+1);
Pm(2:end)= P_minus;
    for i=1:K
        if Pm(i)<R1(j) && R1(j)<Pm(i+1)
            SC(i,j+1)=SC(i,j+1)-1;
        end %end removal loop
    end
            end



        if mod(j,nplot)==0
            A(:,counter)=Atemp(:,2);
            B(:,counter)=Btemp(:,2);
            C(:,counter)=Ctemp(:,2);
            counter=counter+1;

        end
      Atemp(:,1)=Atemp(:,2);
      Btemp(:,1)=Btemp(:,2);
      Ctemp(:,1)=Ctemp(:,2);
end %end iterating difference equations

end

function [Prob] = prob_distribution_reproduction(Nsc, K, r, n, mu)
%starting with allele distribution n generate the probability of each of K
%subtypes reproducing
%mu= K*K mutation matrix
%r=1*K matrix
%n=K*1 matrix

if (nargin==0)
  Nsc=30;
  K=3;
  r=ones(1,K);
  n=10*ones(K,1);
  mu=eye(K);
  u=0.1;
 mu(1,1)=1-u;
  mu(1,2)=u;
```

```
  mu(2,2)=1-u;
  mu(2,3)=u;
end

phi=(r*n); %mean fitness
Prob=zeros(K,K);
P=1;
fitness=mu'*(n.*r'); %sum(mu_(k,i)*r_k*n_k) k=1..K
Prob=P*fitness';
Prob=Prob/phi; %divide by mean fitness
```

```
function beta = birth(x,b,rho,w)
if (nargin<1)
x = 0:0.01:10;
 b= 2;
 rho=5;
 w=5;
end
beta = -b/2*tanh(rho*(x-w))+b/2;
```

Model II (Variable Stem Cell Population Size)

This code simulates trajectories for model with a variable stem cell population size with and without dedifferentiation.

```
%make this function return Teps if you want to find first time when stem
%cell population exceeds a pre-determined threshold
%make the function return Tmut if you re interested in first time 2-mutant
%occurs.
function [Teps]=age_progression_dedifferentiation_variable(u0,epsilon0,Hill,eta)
if (nargin==0)
    u0=0.001 ; %mutation rate for the Moran model (per Moran timestep/ cell division)
      epsilon0=10 %de-differentiation rate
        Hill=1 %Hill coefficient for probability
        eta=0. %eta is prob of asymmetric division
     % s=0.01 ;
     %proliferation advantage of differentiated mutant cells (mutant stem cells are neutral)
end
Teps=0;
Tmut=1;
%time step at which fixation of 2 mutant stem cells occurs

%physical parameters
L=20; %length of domain (age distribution of differentiated cells)
Tgen=5; %timescales on which generation of stem cells turns over
u=u0; %constant mutation rate

%numerical parameters
numx = 101;   %number of grid points in x
dx = L/(numx - 1);
```

```
x = 0:dx:L;   %vector of x values, to be used for plotting
%N=length(x);
dt = 0.05;
CFL=1*dt/dx;
 numt=100/u;

% matrices for output
%initialize everything to zero
A = zeros(numx,3001); %wild-type differentiated cells
B = zeros(numx,3001); %1-mutation differentiated cells
C = zeros(numx,3001); %2-mutation differentiated cells
time = zeros(1,3001); %chronological time
dediff_pop=0; %initialize de-differentiated progenitor cell population

% matrices for computation (get overwritten)
Atemp = zeros(numx,2); %normal cells
Btemp = zeros(numx,2); %mutant 1 cells
Ctemp = zeros(numx,2); %mutant 2 cells
tt=zeros(numt,1);

%biological stem cell parameters
Nsc=100; %total number of  stem cells
K=3;
 % number of subpopulations based on number of mutations cells are allowed to accumulate
fitness=ones(1,K); %fitness of different stem cell mutants
%fitness=[1, 1.1, 1.2];  %fitness of different stem cell mutants
%n=[Nsc, 0, 0]'; %initial number of normal and mutant stem cells

n=[Nsc, 0, 0]'; %initial number of normal and mutant stem cells
KP=Nsc*ones(K,1);
SC=zeros(K,numt+1); %intialize stem cell numbers
SC(:,1) =n; %set stem cell population to initial condition

%initial prob matrix of producing diff. number of offspring
Prob(3,:)=(1-eta)*KP.^Hill./(KP.^Hill+sum(n).^Hill);
Prob(2,:)=eta ;
Prob(1,:)=(1-eta)*sum(n).^Hill./(KP.^Hill+sum(n).^Hill);

%generate random numbers in advance of simulation
R1=rand(1,numt);
R2=rand(1,numt);

% specify proliferation and death rate of differentiated cells
beta=zeros(K,length(x)); %initialize birth rate matrix
bb=zeros(K,length(x)); %initialize birth rate matrix
%r=zeros(K,length(x)); %initialize growth rate
dd=1.; %initialize death rate

%set basal birth rate
```

```
b_diff=[1.5, 1.7, 1.9]; %increasing proliferation of mutant cells
%b_diff=[1.5, (1+s)*1.5, (1+s)^2*1.5] %baseline times selection
%coefficient
for i=1:K
bb(i,:)=fitness(i)*birth(x,b_diff(i),2,5);
end
mu=dd*ones(K,numx); %constant death rate for simplicity
    beta(1:end-1,:)=(1-u)*bb(1:end-1,:);
    beta(end,:)=bb(end,:);
    r=beta-mu;

%specify initial conditions
Atemp(1:end,1) = 0;
A(:,1) = 0;
Btemp(1:end,1) = 0;
B(:,1) = 0;
Ctemp(1:end,1) = 0;
C(:,1) = 0;
time(1)=0;

%BC at x=0 at t=0 (needs to be updated at each time step)
Atemp(1,1)=n(1);
Btemp(1,1)=n(2);
Ctemp(1,1)=n(3);
% total=0; %total mutated cells

%iterate difference equations
for j=1:numt
%return Teps, time when deterministic growth takes over
    if SC(3,j)>= 10*Nsc && SC(3,j-1)<10*Nsc
        Teps= sum(tt(1:j) )
        %returns time point when 2-mutant stem cell pop is 10 times larger than initial Nsc.
    break
    end

    %Uncomment this and return Tmut, first occurence of mutant
% if SC(1,j)==0 && SC(2,j)==0 && SC(3,j)>0 && Tmut==1
%     Tmut=sum(tt(1:j) )
%     %break
% end

    dt = Tgen/sum(SC(:,j));
    %update dt for PDE simulation based on current stem cell pop size
    tt(j)=dt; %update time matrix
    CFL=1*dt/dx;
    if CFL>0.9  %exit if CFL condition for PDE not satisfied
        break
    end
```

```
        %update boundary condition for PDE SC*(2* Prob(2,:)+ Prob(1,:))
        Atemp(1,2)=( Prob(2,1)+ 2*Prob(1,1))*SC(1,j);
        %BC at x=0 updated at each timestep
        Btemp(1,2)=( Prob(2,2)+ 2*Prob(1,2))*SC(2,j);
         %BC at x=0 updated at each timestep
        Ctemp(1,2)=( Prob(2,3)+ 2*Prob(1,3))*SC(3,j);
        %BC at x=0 updated at each timestep


        for i=2:numx %solve equations for aging of differentiated cells by upwinding
            Atemp(i,2) = Atemp(i,1)
            + CFL*(Atemp(i-1,1) -Atemp(i,1))+dt.*r(1,i)*Atemp(i,1);
            Btemp(i,2) = Btemp(i,1)
            + CFL*(Btemp(i-1,1) -Btemp(i,1))+dt.*r(2,i)*Btemp(i,1)+dt*u*bb(1,i)*Atemp(i,1);
            Ctemp(i,2) = Ctemp(i,1)
             +CFL*(Ctemp(i-1,1) -Ctemp(i,1))+dt.*r(3,i)*Ctemp(i,1)+dt*u*bb(2,i)*Btemp(i,1);
        end %end looping over all ages


            lambdap=epsilon0*dt*trapz(x,Ctemp(:,2));
            dediff_pop=[0,0,poissrnd(lambdap)];
            new_pop=SC(:,j)+(dediff_pop)';


Prob(3,:)=(1-eta)*KP.^Hill./(KP.^Hill+sum(new_pop).^Hill);
Prob(2,:)=eta ;
Prob(1,:)=(1-eta)*sum(new_pop).^Hill./(KP.^Hill+sum(new_pop).^Hill)
            %run Moran model with updated total stem cell pop size
            BC=moran_variable_pop_size(new_pop, sum(new_pop) ,K,Prob,2,u0,KP, Hill);
            %call moran_variable_pop_size to calculate new population size
            SC(:,j+1) = BC(:,end);

            Atemp(:,1)=Atemp(:,2);
            Btemp(:,1)=Btemp(:,2);
            Ctemp(:,1)=Ctemp(:,2);
end %end PDE solver

function [trajectory] = moran_variable_pop_size(initsize, N,K,prob,numt,u,KP,Hill)


if (nargin==0)
  N=100;
  K=3;
  prob=zeros(3,K);
  numt=N*10;
  Hill=1;
KP=sum(initsize)*ones(K,1) %carrying capacity set to initial population size
  u=0.01;  %mutation rate
end
```

```
 %set-up mutation matrix
 mu=eye(K);
 mu(1,1)=1-u;
  mu(1,2)=u;
  mu(2,2)=1-u;
  mu(2,3)=u;
  r=ones(1,K); %initialize fitness
  for jj=1:K
      r(jj)=exp(prob(3,jj)-prob(1,jj));
  end

       if (sum(initsize) ~= N)
    error('Initial conditions do not sum up to N');
      end

trajectory=zeros(K,numt);

x=initsize;
z=1; %initialize numt counter
trajectory(:,1)=x;
  tot(1)=N;
R1=rand(1,numt);
R2=rand(1,numt);
eta=0;

while  (z<numt && all(x)>=0)
        %remove one member of population proportional to frequency of the allele
    P_minus=cumsum(x/N);
    Pm=zeros(1,length(P_minus)+1);
Pm(2:end)= P_minus;
    for i=1:K
        if Pm(i)<R1(z) && R1(z)<Pm(i+1)
            x(i)=x(i)-1;
             N=N-1;
        end %end removal loop
    end

     P_plus=cumsum(prob_distribution_reproduction(N, K, r, x, mu) );
    Pp=zeros(1,length(P_plus)+1);
    Pp(2:end)= P_plus;
    for i=1:K
        if Pp(i)<R2(z) && R2(z)<Pp(i+1)
                %new=1; %produce 1 offspring
                new=offspring(1,prob(:,i));
      x(i)=x(i)+new;
      N=N+new;
        end %end reproduction loop
    end
   z=z+1;
```

```
   trajectory(:,z)=x;
  tot(z)=N;

              %update logistic process
        prob(3,:)=(1-eta)*KP.^Hill./(KP.^Hill+N.^Hill);
    prob(2,:)=eta ;
       prob(1,:)=(1-eta)*N.^Hill./(KP.^Hill+N.^Hill);
end; %end while
function nu=offspring(k,p)

z=[cumsum(p)];
n=length(p);        % nmb of possible offspring
offmu=dot(0:n-1,p); % offspring mean
u1=sort(rand(1,k));

 for j=1:n
 u2(j)=length(find(u1<z(j)));
 end

u2=diff([0 u2]);
nu=u2*(0:n-1)';
```
